# A Novel Machine Learning Approach for Predicting Prognosis of SFTS Patients in the Early Stages of Disease

**DOI:** 10.1155/cjid/5389795

**Published:** 2026-06-30

**Authors:** Siqi Li, Ting Wang, Rujia Chen, Yun Wang, Renren Ouyang, Wei Wei, Feng Wang, Shiji Wu, Hongyan Hou

**Affiliations:** ^1^ Department of Laboratory Medicine, Tongji Medical College, Tongji Hospital, Huazhong University of Science and Technology, Wuhan, Hubei, China, hust.edu.cn

**Keywords:** machine learning, prognosis prediction, severe fever with thrombocytopenia syndrome

## Abstract

**Objective:**

Severe fever with thrombocytopenia syndrome (SFTS) is an emerging tick‐borne disease characterized by high morbidity and mortality rates. Timely detection and prognosis prediction are critical for implementing effective clinical interventions. This study aimed to develop a binary classification machine learning model utilizing early clinical and laboratory indicators to predict the prognosis of SFTS patients, facilitating early clinical decision‐making.

**Methods:**

We conducted a retrospective study including 233 SFTS patients diagnosed from October 2021 to May 2024. Clinical and laboratory data at initial diagnosis were collected and subjected to baseline analysis and correlation analysis to identify significant indicators. Using the area under the receiver operating characteristic curve as an indicator of model performance, select the analytical model among machine learning (LR) models, XGBOOST, LightGBM, and random forest. A binary classification machine learning model for predicting survival outcomes was constructed using a logistic regression algorithm in conjunction with identified metrics. The model’s performance was evaluated using the area under the receiver operating characteristic curve (AUC), sensitivity, specificity, accuracy, positive predictive value (PPV), and negative predictive value (NPV). The model was externally validated using a separate cohort of 73 patients.

**Result:**

A total of 233 patients were included in this study, among whom 146 (62.7%) survived and 87 (37.3%) died, with 208 assigned to the internal cohort (177 to the training set and 31 to the test set) and the external validation cohort consisted of 73 patients, including 52 survivors (71.2%) and 21 mortality cases (28.8%). Based on the AUC, the LR model (0.750) is selected, where the values of XGBOOST, LightGBM, and random forest are 0.649, 0.661, and 0.716, respectively. The logistic regression model, incorporating age, lactate dehydrogenase (LDH), albumin, activated partial thromboplastin time (APTT), and platelet count, demonstrated robust predictive accuracy with an AUC of 0.913 (95% CI: 0.821–1.000) for the test set and 0.923 (95% CI: 0.842–1.000) for the external validation set. The model exhibited an accuracy of 0.863, with a sensitivity of 0.952 and an NPV of 0.977. The specificity and PPV were 0.827 and 0.690, respectively. These results indicate that the model maintains robust discriminative performance in an independent cohort, suggesting its potential utility as a screening tool with high sensitivity and favorable NPV.

**Conclusion:**

The model constructed using the first diagnostic indicators described above can accurately determine the patient’s prognosis, which can help the clinician intervene earlier as well as take the necessary life‐saving measures, and has the potential to improve the survival rate of SFTS patients.

**Summary**
• A binary classification machine learning model was developed to predict the prognosis of patients with SFTS using early clinical and laboratory indicators.
• The model was trained on 233 SFTS patients and included variables such as age, LDH, albumin, APTT, and platelet count.
• The logistic regression model demonstrated a strong predictive accuracy with an AUC of 0.913 for the test set and 0.923 for the external validation set.
• The model showed high sensitivity and negative predictive value (NPV), indicating its effectiveness in identifying patients at risk of mortality.
• External validation confirmed the model’s robustness and generalizability, with high sensitivity and NPV, and moderate specificity.
• The study suggests that the integration of machine learning with clinical and laboratory data could improve early clinical decision‐making and potentially enhance the survival rate of SFTS patients.

## 1. Introduction

Severe fever with thrombocytopenia syndrome (SFTS) is an emerging tick‐borne disease caused by SFTS virus (SFTSV), a novel pathogen that belongs to the Phlebovirus genus of Bunyaviridae family [1, 2]. Since its first report in China in 2009, SFTS has emerged in other East Asian regions, such as Japan, Republic of Korea, and Vietnam [3]. By the year 2020, reports of SFTS had spread across 24 provinces in Mainland China, with the infection toll surpassing 13,000 individuals, thereby escalating to a significant public health concern. In 2017, the World Health Organization prioritized SFTS for research due to its potential to cause a pandemic and the absence of a definitive medical treatment [4].

The typical clinical manifestation of SFTS includes fever, leukopenia, and thrombocytopenia, often accompanied by gastrointestinal, respiratory, and neurologic symptoms [1]. In severe cases, patients may exhibit progressive hemorrhagic manifestations, neurologic symptoms, disseminated intravascular coagulation (DIC), and even multiple organ failure (MOF), with a mortality rate of 15%–30% [5, 6]. The clinical features of SFTS are marked by a characteristic set of laboratory abnormalities. The viral load of SFTSV peaks 7–10 days postonset, coincident with the appearance of leukopenia and thrombocytopenia, and a prolonged activated partial thromboplastin time (APTT). Elevated serum levels of alanine aminotransferase (ALT), aspartate aminotransferase (AST), lactate dehydrogenase (LDH), and creatine kinase are indicative of hepatic and muscular involvement. The presence of elevated cytokines, including interleukin‐6 (IL‐6), tumor necrosis factor‐alpha (TNF‐α), and interleukin‐10 (IL‐10), suggests a dysregulated immune response that may contribute to the pathogenesis of SFTS [7, 8]. Our previous studies demonstrated that invasive pulmonary aspergillosis (IPA) occurs in 20%–32% of patients hospitalized for SFTS, which can exacerbate patient morbidity and complicate clinical management [9, 10]. In addition, SFTS and hemophagocytic lymphohistiocytosis (HLH) share clinical similarities, notably the occurrence of a cytokine storm and elevated ferritin levels [4, 11, 12]. The presence of HLH significantly exacerbates the clinical course of SFTS, highlighting the urgency for early recognition and management of this critical complication.

The accurate prediction of patient outcomes is a critical component in the clinical management of SFTS. Currently, the predictive models for SFTS prognosis are suboptimal, often relying on a combination of clinical symptoms and laboratory findings that may not fully capture the complexity of the disease. This has led to a need for more sophisticated analytical approaches that can integrate multiple data points and identify patterns that are indicative of disease severity and prognosis. In response to this challenge, machine learning (ML) has been used as a means to enhance the predictive accuracy of prognostic models [13].

## 2. Materials and Methods

### 2.1. Study Design and Population Cohorts

This study was conducted retrospectively, with clinical data collected from two medical centers in Wuhan, China: Tongji Hospital Qiaokou Branch in Hankou District (Cohort 1) and Tongji Hospital Optics Valley Branch in Wuhan East Lake High‐Tech Development Zone (Cohort 2).

A total of 233 patients with an initial diagnosis of SFTS from October 2021 to May 2024 were included in this study. Cohort 1 (modeling and internal validation): patients with an initial diagnosis of SFTS from October 2021 to October 2023 were included. Furthermore, for the purpose of external validation, we included 73 patients with SFTS who were first diagnosed at Tongji Hospital during the period from February to October 2024 in Cohort 2.

Within 24 h of admission, the basic clinical information including age, gender, underlying diseases, medical history, and all initial laboratory results of the research subjects in the medical record system, including blood cell count, liver function tests, renal function tests, coagulation function, inflammatory markers, and cytokines, were collected.

Routine blood tests were performed using the Xisenmechem XN9000. Coagulation function was tested using a Stago fully automated hemagglutination analyzer, and liver and kidney function levels were tested using a Roche Cobas e701 automated electrochemiluminescence immunoassay system.

The primary outcome of this study was defined as a binary endpoint of short‐term prognosis, categorized as survival versus mortality within 28 days following hospital admission. Patients who died within 28 days were assigned to the mortality group, whereas those who survived beyond 28 days were classified as the survival group. All patients were followed for at least 28 days after admission to ensure complete outcome assessment. No patients were lost to follow‐up or excluded due to missing outcome data. The 28‐day observation window was selected as it represents a widely accepted clinical endpoint in studies of severe infectious diseases, allowing standardized evaluation of short‐term mortality and facilitating comparison with prior research.

### 2.2. Model Fitting and Evaluation

The ML model was developed incorporating variables that demonstrated statistical significance, along with factors highlighted as important in the existing literature. To reduce multicollinearity, a correlation analysis was performed to identify indicators that were highly correlated. Subsequently, receiver operating characteristic (ROC) analysis was used to identify significant features of the indicators.

The dataset from Cohort 1 was randomly divided into a training set (85%) and an independent internal test set (15%). Model development was conducted exclusively within the training dataset.

To ensure model robustness and reduce variability, fivefold cross‐validation was performed using a stratified random sampling strategy, in which the dataset was partitioned into five approximately equal subsets while preserving the proportion of outcome events in each fold. In each iteration, fourfold were used for model training and the remaining fold was used for validation. This process was repeated five times so that each fold served once as the validation set. The average performance across the fivefold was used to guide model selection and optimization.

Given the relatively modest sample size, extensive hyperparameter tuning was not performed in order to minimize the risk of overfitting. For logistic regression (LR), L2 regularization with default parameter settings was applied. LR is a stable and low‐variance algorithm, particularly appropriate for small clinical datasets.

For tree‐based models (XGBoost, LightGBM, and random forest), default parameter settings were used to ensure a consistent and unbiased comparison across algorithms. This strategy was adopted to prioritize model stability and generalizability rather than aggressive optimization.

The internal test set was not involved in feature selection, model training, or hyperparameter tuning and was used only once after model finalization to obtain an unbiased estimate of model performance.

External validation was subsequently performed using an independent cohort (Cohort 2), collected from a separate hospital branch during a different time period, to further evaluate model generalizability.

LR is a statistical learning method used to solve classification problems. It is a widely used ML algorithm, especially in binary classification problems. SHAP (Shapley Additive Explanations) analysis was performed using the SHAP LinearExplainer, which is specifically designed for linear models, to quantify feature contributions and enhance interpretability of the LR model.

**FIGURE 1:**
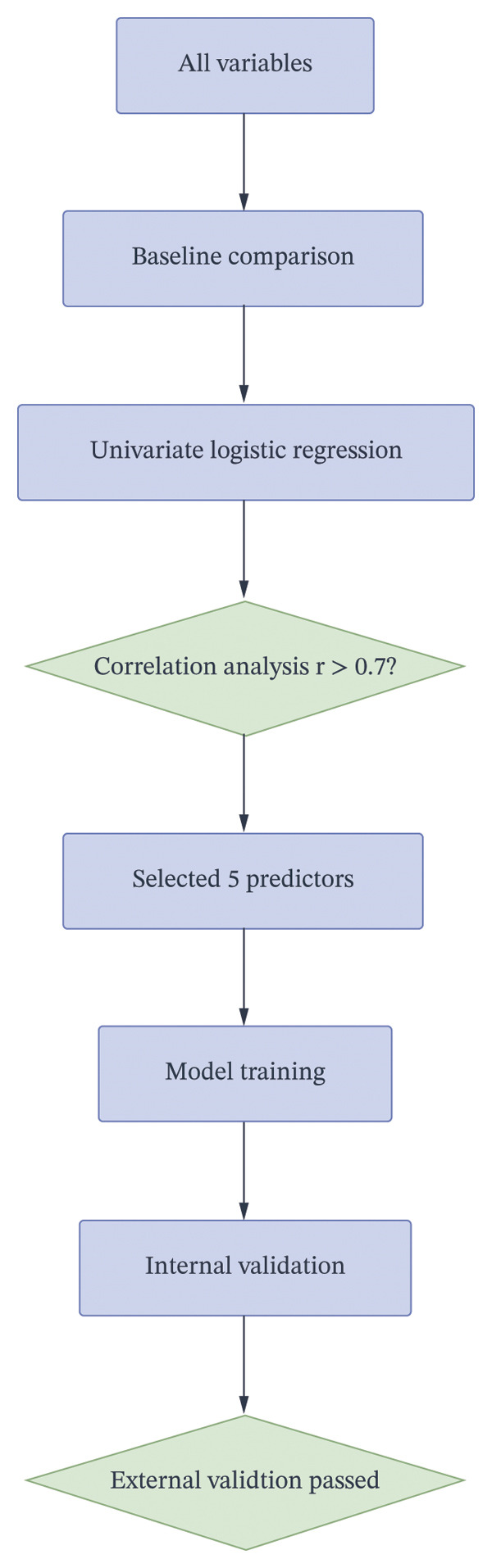
Workflow of feature selection and model development. All candidate variables underwent baseline comparison and univariate logistic regression analysis. Correlation analysis was subsequently performed to reduce multicollinearity, and five predictors were ultimately selected for model construction.

Unlike traditional regression‐based modeling, excessive univariate prefiltering was avoided to preserve potentially informative predictors for ML algorithms. The overall feature selection and model development process is illustrated as a structured workflow in Figure 1 to enhance methodological transparency and reproducibility.

Model selection was guided not only by cross‐validated AUC but also by considerations of interpretability, stability, and overfitting risk. Model performance was evaluated in terms of discrimination and calibration. Calibration was assessed using the calibration slope and calibration curves. A cutoff value of 0.4 was used to stratify patients into high‐ and low‐risk groups based on ROC analysis. Patients were stratified into high‐risk and low‐risk groups based on a cutoff value of 0.4 applied to the predicted probabilities.

**FIGURE 2:**
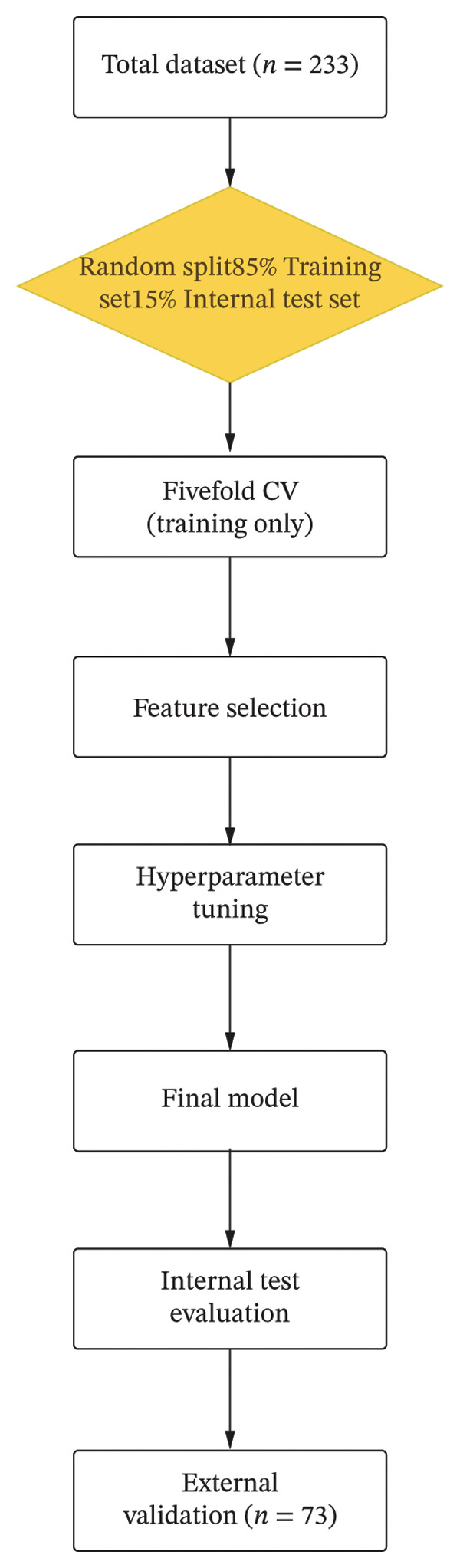
Workflow of dataset splitting, model training, and validation. The total dataset (*n* = 233) was randomly divided into a training set (85%) and an internal test set (15%). Fivefold cross‐validation was performed within the training set for feature selection and hyperparameter tuning. The final model was subsequently evaluated using the internal test set and an independent external validation cohort.

A detailed methodological workflow diagram summarizing data preprocessing, missing data handling, feature selection, model development, and validation procedures is presented in Figure 2.

### 2.3. Statistics

Continuous variables are presented either as the mean with standard deviation (mean ± SD) or as the median with interquartile range (median [IQ]), based on the distribution of the data. For comparing variables across groups, we employed Student’s *t*‐test, the Mann–Whitney *U* test, or the one‐way ANOVA, depending on the data’s characteristics. Categorical variables were compared using the *χ* ^2^ test or Fisher exact test based on sample size and expected cell frequency. A ROC curve analysis was performed to determine the optimal cutoff values for parameters to achieve the highest sensitivity and specificity. These tests were used to analyze associations between categorical variables in the study. *p* < 0.05 was considered a statistically significant difference.

### 2.4. Missing Data Handling

Missing values were assessed prior to model construction. For variables with less than 5% missingness, mean or median imputation was applied depending on distribution characteristics.

Several laboratory variables, particularly cytokines (IL‐6, IL‐10, and TNF‐α) and pancreatic enzymes (amylopsin and lipase), exhibited relatively high missing rates (> 25%). These indicators were not routinely measured at initial presentation and were ordered selectively based on clinical judgment. Therefore, missingness was primarily attributable to clinical testing practices rather than outcome‐dependent mechanisms.

The distribution of missing values was examined between survival and mortality groups, and no statistically significant group‐dependent differences were observed.

Although multiple imputation techniques were considered, advanced imputation was not performed due to the relatively high missingness rate in certain variables and the modest overall sample size. Imputing variables with substantial missing proportions may increase model instability and reduce interpretability. Therefore, variables with high missing rates were excluded from model development, and only indicators with complete or near‐complete data were retained for feature selection and modeling to enhance model stability and clinical applicability.

### 2.5. Ethics Statement

The study was conducted in accordance with institutional ethical guidelines, and patient confidentiality was strictly maintained.

## 3. Results

### 3.1. Basic Characteristics of the Cohort of Patients With Different Prognoses Included

Comparing the survival and mortality groups within Cohort 1, we identified no significant differences in gender, clinical symptoms, comorbidities, and treatment approaches. The survival group comprised 54.08% females and 45.92% males, while the mortality group had a similar distribution with 50.58% females and 49.43% males.

The most common clinical manifestation was fever (91.85%). Clinical manifestations of weakness were observed in 28.76% of individuals, with muscle pain affecting 8.58% and nausea and vomiting occurring in 24.03% and 30.04% of cases, respectively. Abdominal pain was reported in 9.01% of patients, and diarrhea was present in 38.20%. In terms of comorbidities, hypertension was noted in 31.76% of the patient population, diabetes mellitus in 8.58%, coronary artery disease in 11.59%, chronic obstructive pulmonary disease (COPD) in 7.73%, and *tuberculosis* in 2.15%. Regarding therapeutic interventions, hormone therapy was prescribed for 29.61% of patients, while 15.02% were treated with immunoglobulin. No significant differences were identified in the prevalence of a history of fieldwork or tick exposure between survivors and nonsurvivors of the disease.

It warrants emphasis that the median age within the survival group was modestly lower, at 61 years, in contrast to the mortality group, where it was 68 years, suggesting a conceivable link between advanced age and an elevated risk of mortality. Furthermore, the survival group exhibited a heightened propensity for symptoms such as impaired consciousness and concurrent HLH, complexities that were more frequently associated with the necessity for advanced therapeutic interventions, including continuous renal replacement therapy (CRRT) and respiratory support, in comparison to the mortality group. This observation may provide critical insights into the clinical profile and treatment requirements of the patient cohort (Table 1).

**TABLE 1:** The demographic and clinical features of SFTS patients on admission.

Parameters	Total (*n* = 223)	Survival (*n* = 146)	Deceased (*n* = 87)	*p* value	Statistical methods
Age, median (Q1–Q3), year	65 (57–71)	61 (55–70)	68 (62–73)	^∗∗∗^	Mann–Whitney U
Sex					Chi‐square test
Female, *n* (%)	126 (54.08)	82 (56.16)	44 (50.58)	ns	
Male, *n* (%)	107 (45.92)	64 (43.84)	43 (49.43)		
Symptoms, *n* (%)					
Fever	214 (91.85)	137 (93.84)	77 (88.51)	ns	Chi‐square test
Weakness	67 (28.76)	38 (26.03)	29 (33.33)	ns	Chi‐square test
Muscular soreness	20 (8.58)	13 (8.90)	7 (8.05)	ns	Chi‐square test
Inappetence	47 (20.17)	29 (19.86)	18 (20.69)	ns	Chi‐square test
Nausea	56 (24.03)	38 (26.03)	18 (20.69)	ns	Chi‐square test
Vomiting	70 (30.04)	46 (31.51)	24 (27.59)	ns	Chi‐square test
Abdominal pain	21 (9.01)	17 (11.64)	4 (4.60)	ns	Chi‐square test
Diarrhea	89 (38.20)	58 (39.73)	31 (35.63)	ns	Chi‐square test
Headache and dizziness	58 (24.89)	36 (24.66)	22 (25.29)	ns	Chi‐square test
Consciousness disorder	70 (30.04)	27 (18.49)	43 (49.43)	^∗∗∗^	Chi‐square test
Comorbidities, *n* (%)					
Hypertension	74 (31.76)	40 (27.40)	34 (39.08)	ns	Chi‐square test
Diabetes mellitus	20 (8.58)	10 (6.85)	10 (11.49)	ns	Chi‐square test
Coronary heart disease	27 (11.59)	15 (10.27)	12 (13.79)	ns	Chi‐square test
COPD	18 (7.73)	10 (6.85)	8 (9.20)	ns	Chi‐square test
Tuberculosis	5 (2.15)	3 (2.06)	2 (2.30)	ns	Chi‐square test
Therapy, *n* (%)					
Corticosteroid	69 (29.61)	38 (26.03)	31 (35.63)	ns	Chi‐square test
CRRT	55 (23.61)	19 (13.01)	36 (41.38)	^∗∗∗^	Chi‐square test
Intravenous immunoglobulin	35 (15.02)	17 (11.64)	18 (20.69)	ns	Chi‐square test
Respiratory support	31 (13.31)	5 (3.43)	26 (29.89)	^∗∗∗^	Chi‐square test
Complicated with HLH, *n* (%)	112 (48.07)	51 (34.93)	61 (70.12)	^∗∗∗^	Chi‐square test
History of tick bite	30 (12.88)	20 (13.70)	10 (11.49)	ns	Chi‐square test
History of field activities	38 (16.31)	23 (15.75)	15 (17.24)	ns	Chi‐square test

*Note:* HLH, hemophagocytic lymphohistiocytosis. The differences of the parameters were compared between the survival and deceased groups.

Abbreviations: COPD, chronic obstructive pulmonary disease; CRRT, continuous renal replacement therapy; Q, quartile; SD, standard deviation.

^∗^Indicates *p* < 0.05.

^∗∗^Indicates *p* < 0.0.

^∗∗∗^Indicates *p* < 0.001.

Regarding laboratory parameters as detailed in Table 2, a number of significant disparities were noted when comparing the two cohorts. The mortality group demonstrated markedly reduced platelet counts, alongside elevated levels of hepatic enzymes, specifically ALT and AST, and increased LDH activity. Furthermore, pronounced variations were identified in coagulation profiles, inflammatory markers, and cytokine levels, potentially mirroring the gravity of the disease and its systemic physiological ramifications.

**TABLE 2:** Comparison of laboratory markers between the surviving and deceased group of SFTS patients upon admission.

Parameters	Total (*n* = 233)	Survival (*n* = 146)	Deceased (*n* = 87)	*p* value	Missing %
Blood routine indicators					
WBC (× 10^9^/L)	5.00 ± 4.86	5.17 ± 4.93	4.80 ± 4.81	Ns	0.00
Neutrophiles (× 10^9^/L)	3.75 ± 4.30	3.92 ± 4.48	3.53 ± 4.10	Ns	0.00
Lymphocytes (× 10^9^/L)	0.94 ± 0.94	1.00 ± 1.02	0.86 ± 0.83	Ns	0.00
Monocytes (× 10^9^/L)	0.35 ± 0.46	0.36 ± 0.42	0.35 ± 0.52	^∗^	0.00
RBC (× 10^12^/L)	4.32 ± 0.76	4.35 ± 0.66	4.30 ± 0.89	Ns	0.00
Hemoglobin (g/L)	130.26 ± 22.70	131.38 ± 21.88	128.84 ± 23.89	Ns	0.00
Platelet (× 10^9^/L)	50.90 ± 35.36	58.21 ± 41.49	41.59 ± 22.78	^∗∗∗^	0.00
Blood biochemistry indicators					
ALT (U/L)	125.70 ± 100.53	101.31 ± 76.89	156.33 ± 117.95	^∗∗^	1.72
AST (U/L)	392.38 ± 432.26	236.85 ± 210.06	587.70 ± 548.85	^∗∗∗^	1.72
LDH (U/L)	1079.29 ± 596.38	866.51 ± 548.96	1324.19 ± 557.62	^∗∗∗^	1.72
ALP (U/L)	127.04 ± 195.02	125.85 ± 252.71	128.53 ± 79.63	^∗∗∗^	1.72
Total protein (g/L)	61.26 ± 6.80	61.79 ± 6.85	60.58 ± 6.77	^∗^	1.72
Albumin (g/L)	32.44 ± 4.83	33.07 ± 4.61	31.65 ± 5.02	^∗^	1.72
Globulin (g/L)	28.82 ± 4.56	28.72 ± 4.48	28.93 ± 4.72	Ns	1.72
TBIL (μmol/L)	14.65 ± 32.47	15.21 ± 42.24	13.93 ± 11.06	^∗∗^	1.72
DBIL (μmol/L)	9.89 ± 26.92	10.31 ± 35.01	9.35 ± 10.02	^∗∗∗^	1.72
IBIL (μmol/L)	4.67 ± 6.42	4.90 ± 7.85	4.40 ± 4.22	Ns	1.72
Urea (mmol/L)	7.57 ± 5.51	6.55 ± 5.24	8.86 ± 5.63	^∗∗∗^	2.58
Uric acid (μmol/L)	281.43 ± 131.66	242.92 ± 77.60	329.79 ± 166.50	^∗∗∗^	2.58
Creatinine (μmol/L)	110.37 ± 90.35	90.31 ± 53.78	135.56 ± 117.65	^∗∗∗^	2.58
HCO3^-^ (mmol/L)	19.55 ± 3.88	20.31 ± 3.42	18.59 ± 4.24	^∗∗^	2.58
eGFR (ml/min/1.73 m^2^)	69.45 ± 26.88	78.22 ± 23.55	58.43 ± 26.99	^∗∗∗^	2.58
Amylopsin (U/L)	116.31 ± 101.03	90.98 ± 55.93	146.85 ± 131.50	Ns	14.16
Lipase (IU/L)	370.54 ± 535.2	201.42 ± 193.42	564.4 ± 712.64	^∗∗^	14.16
Triglyceride (mmol/L)	2.28 ± 1.29	2.33 ± 1.27	2.22 ± 1.32	Ns	27.46
Total cholesterol (mmol/L)	3.02 ± 0.91	3.08 ± 0.86	2.94 ± 0.98	^∗^	1.29
Coagulation markers					
TT (S)	50.52 ± 59.96	36.23 ± 45.33	67.25 ± 70.44	^∗∗∗^	8.15
PT (S)	17.06 ± 19.67	17.53 ± 21.92	16.52 ± 16.89	^∗∗∗^	8.15
APTT (S)	70.13 ± 37.43	56.76 ± 23.10	85.13 ± 44.44	^∗∗∗^	8.15
Fibrinogen (g/L)	2.66 ± 0.82	2.81 ± 0.89	2.50 ± 0.71	^∗∗^	8.15
D‐dimer (μg/mL FEU)	6.41 ± 6.42	4.18 ± 5.18	8.64 ± 6.82	^∗∗∗^	9.44
Inflammatory indicators					
hsCRP (mg/L)	14.56 ± 23.38	11.02 ± 22.17	18.98 ± 24.38	^∗∗∗^	9.01
Ferritin (μg/L)	23311.45 ± 19862.65	14547.50 ± 16848.72	32974.27 ± 18559.62	^∗∗∗^	28.76
PCT (ng/mL)	1.22 ± 3.93	0.57 ± 1.91	2.17 ± 5.63	^∗∗∗^	13.30
Cytokine indicators					
IL‐1β (pg/mL)	11.08 ± 20.85	7.26 ± 9.26	17.95 ± 31.72	^∗∗^	27.04
IL‐2R (U/mL)	1564.65 ± 1114.80	1201.62 ± 628.56	2036.60 ± 1410.23	^∗∗∗^	27.04
IL‐6 (pg/mL)	258.13 ± 698.08	51.84 ± 85.72	499.77 ± 977.19	^∗∗∗^	14.59
IL‐8 (pg/mL)	271.23 ± 966.78	32.82 ± 49.77	571.16 ± 1407.04	^∗∗∗^	27.04
IL‐10 (pg/mL)	128.55 ± 190.78	66.98 ± 106.24	213.95 ± 244.53	^∗∗∗^	27.04
TNF‐α (pg/mL)	75.80 ± 158.70	27.32 ± 37.66	138.82 ± 223.45	^∗∗∗^	27.04

*Note:* ALP, alkaline phosphatase; ALT, alanine aminotransferase; AST, aspartate aminotransferase; DBIL, direct bilirubin; IBIL, indirect bilirubin; LDH, lactate dehydrogenase; PCT, procalcitonin; TBIL, total bilirubin; The data were shown as mean and standard deviation. Continuous variables were compared using Student’s *t*‐test or Mann–Whitney *U* test, after assessing the normal distribution of the supplementary data.

Abbreviations: APTT, activated partial thromboplastin time; eGFR, estimated glomerular filtration rate; hsCRP, high‐sensitivity C‐reactive protein; PT, prothrombin time; RBC, red blood cell; TNF‐α, tumor necrosis factor‐alpha; TT, thrombin time; WBC, white blood cell.

^∗^Indicates *p* < 0.05.

^∗∗^Indicates *p* < 0.01.

^∗∗∗^Indicates *p* < 0.001.

### 3.2. Model Screening

In accordance with a stringent feature selection protocol delineated within the Materials and Methods section of this report, we have ascertained a set of pivotal features with the potential to serve as prognostic biomarkers for SFTS. The process of feature selection was meticulous, ensuring that the identified metrics would be the most influential in predicting severe SFTS outcomes. Five key parameters—age, LDH, APTT, platelet count, and albumin—were ultimately selected for the development of a ML model.

**FIGURE 3:**
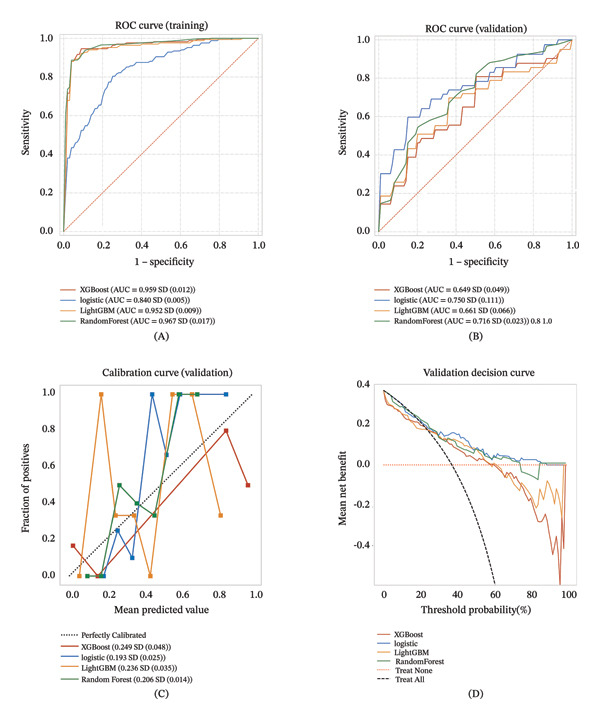
Model performance metrics. (A) Calibration curve comparison for various machine learning models. (B) ROC curves for the training phase, illustrating the sensitivity and specificity balance for XGBoost, logistic regression, LightGBM, and random forest models, with AUC values indicating their discriminatory power. (C) ROC curves for the validation phase. (D) Decision curve analysis for the validation phase.

Employing these metrics, a comparative analysis was conducted to determine the optimal LR model. This model was evaluated against other classification models, including XGBOOST, LightGBM, random forest, and standard logistic classification algorithms. The LR model demonstrated superior predictive accuracy for severe SFTS cases and was thus chosen for its predictive efficacy. The performance of the selected model is illustrated in Figure 3, which provides a visual representation of its predictive capabilities when applied to the identified metrics.

### 3.3. Performance of the LR Model in Predicting Survival and Mortality

In the context of prognostication for patients afflicted with SFTS, the LR model exhibited robust predictive performance. The area under the receiver operating characteristic curve (AUC) for the LR model was calculated to be 0.750, with a 95% confidence interval (CI) ranging from 0.533 to 0.955. The model demonstrated an accuracy of 0.726, accompanied by a 95% CI extending from 0.647 to 0.805, thereby signifying a high degree of correctness in its predictions.

Furthermore, the model’s sensitivity was determined to be 0.736, with a 95% CI of 0.595–0.877, indicating its ability to correctly identify patients at risk of mortality. Meanwhile, the specificity of the model was 0.719 (95% CI: 0.581–0.857), reflecting its ability to accurately classify patients who survived.

Additionally, the positive predictive value (PPV) of the LR model was 0.628 (95% CI: 0.503–0.753), indicating the proportion of patients predicted to be at high risk who subsequently experienced mortality. The negative predictive value (NPV) was 0.830 (95% CI: 0.761–0.899), indicating the proportion of patients predicted to be at low risk who survived. These metrics are detailed in Table 3.

**TABLE 3:** Multimodel performance comparison.

Classification model	AUC (95% CI)	Cutoff (95% CI)	Accuracy (95% CI)	Sensitivity (95% CI)	Specificity (95% CI)	Positive predictive value (95% CI)	Negative predictive value (95% CI)
XGBoost	0.649 (0.387–0.911)	0.837 (0.808–0.866)	0.672 (0.592–0.753)	0.314 (0.096–0.532)	0.889 (0.796–0.981)	0.680 (0.402–0.958)	0.693 (0.608–0.779)
Logistic	0.750 (0.533–0.955)	0.331 (0.289–0.374)	0.726 (0.647–0.805)	0.736 (0.595–0.877)	0.719 (0.581–0.857)	0.628 (0.503–0.753)	0.830 (0.761–0.899)
LightGBM	0.661 (0.402–0.921)	0.553 (0.479–0.626)	0.674 (0.614–0.733)	0.500 (0.466–0.534)	0.774 (0.692–0.857)	0.581 (0.500–0.662)	0.721 (0.681–0.762)
Random forest	0.716 (0.485–0.947)	0.590 (0.512–0.668)	0.673 (0.634–0.713)	0.331 (0.167–0.494)	0.873 (0.745–1.001)	0.733 (0.512–0.955)	0.692 (0.662–0.722)

Abbreviation: AUC, area under the curve.

**FIGURE 4:**
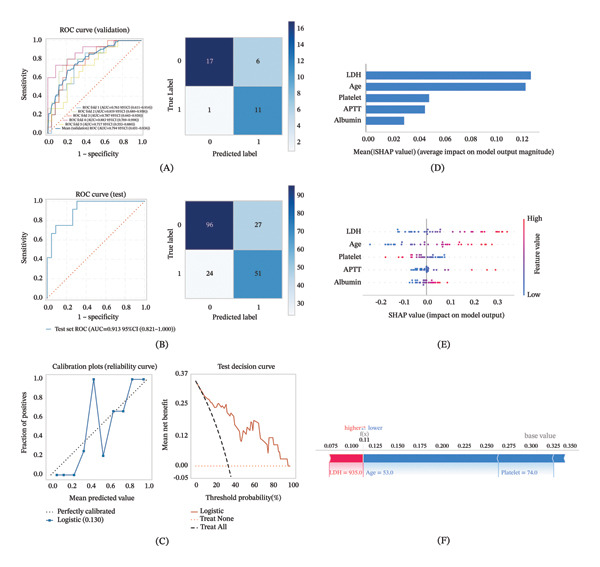
Model calibration, ROC curves, and SHAP values. (A) Calibration plot (reliability curve) of the logistic regression model. (B) Receiver operating characteristic (ROC) curve for the test cohort. (C) Decision curve analysis (DCA) demonstrating net benefit across a range of threshold probabilities. (D) SHAP summary plot illustrating the average contribution of key features (albumin, APTT, platelet, age, and LDH) to model output. (E) SHAP dependence plots showing the relationship between individual feature values and predicted risk. (F) Confusion matrix displaying predicted versus observed outcomes.

The convergence of the confusion matrices from the training and validation sets, as illustrated in Figure 4A,B, offers a clear depiction of the model’s predictive performance. Figure 4C further demonstrates the model’s prognostic performance using a validation ROC curve, illustrating the trade‐off between sensitivity and specificity across different thresholds, while Figure 4D–F provides insights into model interpretability through SHAP analysis. Notably, age and LDH are identified as key parameters with significant influence on the model’s output, as indicated by their pronounced SHAP values, whereas APTT, platelet count, and albumin exhibit comparatively lower SHAP values, implying a more modest impact on the predictive accuracy. The collective analysis, including the ROC curve validation and SHAP value interpretation, provides a comprehensive understanding of the model’s clinical utility and its capacity to inform patient risk stratification in SFTS.

### 3.4. External Validation

The independent validation subset (Cohort 2) was temporally and geographically distinct from the training dataset and was not involved in any stage of model development, thereby minimizing the risk of information leakage. The predictive performance of the model was externally validated using this independent cohort. Using a predefined cutoff value of 0.4 for risk stratification, patients with predicted probabilities ≥ 0.4 were classified as high‐risk patients, whereas those with probabilities < 0.4 were considered low‐risk patients. The model achieved an AUC of 0.923. At this threshold, the model demonstrated an accuracy of 0.863, with a sensitivity of 0.952 and a specificity of 0.827, indicating good overall classification performance. The PPV was 0.690, while the NPV reached 0.977, as shown in Table 4. Although the model exhibited strong discriminative ability, the results should be interpreted with caution due to the relatively limited sample size of the external validation cohort.

**TABLE 4:** External validation of performance.

AUC	Cutoff	Accuracy	Sensitivity	Specificity	PPV	NPV
0.923	0.400	0.863	0.952	0.827	0.690	0.977

**FIGURE 5:**
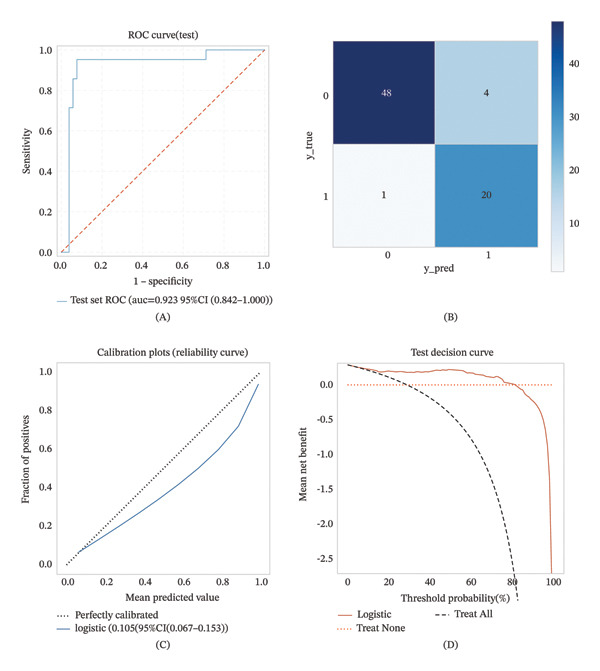
Test decision curve and ROC curve. (A) Test decision curve, presenting the mean net benefit of the logistic model at various threshold probabilities. (B) ROC curve for the test set. (C) Calibration plot (reliability curve) for the logistic model. (D) Graphical representation of true versus predicted outcomes.

Figure 5 presents a comprehensive graphical analysis of the model’s performance on the test set. The ROC curve (Figure 5, test set ROC), with an AUC of 0.923 (95% CI: 0.842–1.000), demonstrates the model’s strong discriminative performance. The decision curve analysis (DCA) depicted in Figure 5 illustrates the net benefit of the model across various threshold probabilities, providing insights into how the model’s predictions impact patient management decisions. Compared with the “Treat All” and “Treat None” strategies, the model demonstrated superior net benefit across a clinically relevant range of threshold probabilities (net benefit can be defined as the net gain at a specific threshold probability, which is the benefit of correctly predicted individuals minus the loss caused by incorrectly predicted individuals). The calibration plot (Figure 5, calibration plots) demonstrates the agreement between the predicted probabilities and observed outcomes, with a calibration slope of 0.105 and a 95% CI from 0.067 to 0.153, suggesting that the model’s predictions are well calibrated.

In summary, the external validation confirms the robustness and generalizability of our predictive model, with high sensitivity and NPV, and moderate specificity, positioning it as a potentially valuable tool for clinical decision‐making in the context of SFTS.

## 4. Discussion

The disease is characterized by the sudden onset of fever and gastrointestinal symptoms, followed by a gradual decline in platelets and white blood cells [14]. The typical course of the infection has four distinct periods, incubation, fever, MOF, and recovery [15]. The fever stage is characterized by the appearance of flu‐like symptoms, and a high viral load can be detected during this stage [14, 16]. It is an important marker for clinical diagnosis. The next stage is characterized by progressive deterioration of MOF in fatal cases [17]. MOF progresses rapidly, first in the liver and heart and then in the lungs and kidneys. MOF may overlap with the febrile phase, during which the serum viral load of the survivor gradually decreases, but still persists for 7–14 days [18]. Serum viral loads remain high in patients who die. Important biomarkers such as AST, creatine kinase, and LDH are significantly higher in fatal cases than in survivors [19].

In the context of SFTS, liver injury is a common complication that is strongly associated with mortality. From the perspective of liver injury, APTT and albumin are two important indices. APTT is a key assay for assessing the function of endogenous and common coagulation pathways. In patients with SFTS associated with liver injury, the liver’s ability to synthesize coagulation factors may be impaired, resulting in a prolonged APTT. This prolongation reflects impaired hepatic synthesis and its effect on hemostasis.

Albumin is synthesized only by the liver and is a key indicator of hepatic synthetic function. In SFTS patients with liver injury, the liver’s ability to produce albumin is reduced, resulting in lower serum albumin levels. Reduced albumin levels not only reflect the severity of hepatic dysfunction but also indicate an impaired ability of the liver to maintain colloid osmolality and other important functions. This may affect the patient’s nutritional status and fluid balance and is associated with mortality. In conclusion, APTT and albumin are important markers for assessing liver injury in SFTS patients. They provide important information for assessing the severity of liver injury, predicting disease progression, and guiding clinical treatment decisions. Close monitoring of these markers can help improve the management of SFTS patients.

SFTS presents with nonspecific clinical manifestations, such as fever and thrombocytopenia, which often overlap with other febrile illnesses, making early clinical identification challenging. Although definitive diagnosis relies on nucleic acid testing, this approach may not provide timely guidance for risk stratification at the initial clinical encounter, potentially delaying appropriate management in high‐risk patients [20–22]. Therefore, there is a critical need for predictive tools that can be applied early in the disease course to identify patients at high risk of mortality. ML models, based on readily available clinical and laboratory indicators, offer a promising approach for early risk stratification, enabling timely intervention and potentially improving survival outcomes in SFTS patients [23, 24].

ML is a rapidly evolving computational approach in the biomedical field. In this study, we integrated ML with clinical and laboratory data from SFTS patients to predict survival versus mortality outcomes. While previous models have often incorporated subjective clinical symptoms alongside laboratory indicators, objective and readily available laboratory parameters have been relatively underutilized. In this study, we propose a novel ML framework based on easily accessible and objective laboratory indicators to enable early risk stratification of SFTS patients. This approach facilitates the early identification of patients at high risk of mortality, thereby supporting timely clinical intervention. Through a comprehensive modeling process, including baseline analysis, feature selection, model comparison, and external validation, five key predictors—age, LDH, APTT, platelet count, and albumin—were identified. Models constructed using these variables demonstrated strong predictive performance.

SHAP is based on cooperative game theory and utilizes patient data to provide deeper insights into model predictions. As interpretable ML evolves, SHAP will continue to play a key role in improving the interpretability and accountability of ML models [25]. The directionality of SHAP contributions was consistent with LR coefficients, reinforcing the internal consistency and interpretability of the model. Compared to traditional odds ratios, SHAP enables patient‐level explanation of predicted risk, facilitating transparent clinical decision support rather than solely population‐level inference.

In addition to this, during the collection of patient metrics, certain metrics may perform well when included in the model, such as the phenomenon of combined hemophilia, cytokine indicators (IL‐6, IL‐10, and TNF‐α), ferritin, etc. As this study focused on predicting survival versus mortality using early clinical indicators, some patients were excluded due to missing laboratory data at initial presentation. The indicators included in this study are routinely available and rapidly obtainable laboratory parameters that are often overlooked in clinical practice. The rational use of these indicators may facilitate early identification of patients at high risk of mortality, thereby supporting timely intervention and potentially improving survival outcomes in SFTS patients.

It is worth noting that several laboratory indicators–including amylopsin, lipase, triglyceride, and inflammatory/cytokine markers such as IL‐6, IL‐10, and TNF‐α—showed a relatively high proportion of missing data (> 14%–28%). These missing values primarily resulted from the unavailability of these tests at initial presentation, as many of them are not part of routine early‐stage diagnostics. Consequently, they were not included in the final model. This limitation may lead to the underestimation of the predictive power of immune or inflammatory factors. However, by focusing on commonly available indicators with minimal missingness, the model maintained its clinical applicability and generalizability. While the exclusion of these variables may limit exploration of immune‐related pathways associated with mortality, prioritizing routinely available indicators enhances the clinical feasibility and reproducibility of the model in real‐world early triage settings, facilitating early identification of patients at high risk of mortality.

As shown in Table 2, indicators such as ferritin and cytokine levels (IL‐6, IL‐10, TNF‐α) demonstrated significant differences between survivors and nonsurvivors (*p* < 0.001), suggesting their potential prognostic relevance. However, due to high missing rates at initial diagnosis, these variables were excluded from model training in the current study.

From a practical clinical workflow perspective, the model is designed for application at the time of hospital admission. All predictors are routinely available laboratory parameters obtained during the initial evaluation. Once laboratory data are available, predicted mortality risk can be calculated automatically. This enables early identification of patients at high risk of mortality and supports timely escalation of monitoring, appropriate referral to intensive care, and optimized allocation of medical resources.

The model demonstrates high sensitivity with moderate specificity. The model demonstrates high sensitivity with moderate specificity. Similar findings have been reported in previous studies on SFTS and other infectious disease prediction models [26]. Compared with previous studies, the present study provides several additional contributions. First, the model was externally validated using an independent cohort that was temporally and geographically distinct from the training dataset, which enhances its generalizability. Second, the model demonstrated high sensitivity and NPV, making it particularly suitable for early risk stratification and clinical triage. Third, the use of routinely available clinical variables improves the feasibility of its implementation in real‐world clinical practice. Given its high NPV, the model may serve as an effective early triage tool to identify high‐risk patients at admission. This may facilitate closer monitoring and timely intervention for high‐risk patients, while allowing low‐risk patients to receive standard management, thereby optimizing resource allocation. In the context of severe infectious diseases such as SFTS, prioritizing sensitivity is clinically justifiable to minimize missed patients at high risk of mortality, which is consistent with previous studies emphasizing early identification of high‐risk patients in severe infectious diseases. While moderate specificity may lead to some false‐positive classifications, the clinical consequence would generally involve closer monitoring rather than unnecessary invasive treatment. Therefore, the trade‐off between sensitivity and specificity may be acceptable in a risk stratification framework.

Given its early availability and reliance on routine laboratory indicators, the model may also function as a triage tool at hospital admission. Patients classified as being at high risk of mortality could be prioritized for closer monitoring or higher‐level care, whereas those at lower risk may continue with standard management. Such risk stratification may enhance workflow efficiency in resource‐limited settings.

Because the model relies exclusively on structured laboratory parameters routinely captured in electronic health record (EHR) systems, automated integration into hospital information systems is technically feasible. Risk scores could be generated automatically once laboratory results are uploaded, without additional manual input, thereby facilitating seamless clinical implementation.

Compared with previously reported prognostic scoring systems, the present model incorporates a limited number of routinely available laboratory variables while maintaining strong discriminative performance. Its relative simplicity and transparent interpretability may facilitate broader clinical adoption, particularly in settings where complex scoring systems requiring numerous or specialized indicators are less practical.

This study acknowledges several limitations inherent to its design and execution. First, the modest size of the dataset employed may have constrained the generalizability of the findings. Second, although external validation was performed using an independent cohort, the lack of multicenter validation with a larger and more diverse population may still introduce potential bias and limit the generalizability of the findings. Third, the analysis in this report primarily focuses on individual indicators without delving into more comprehensive or multidimensional assessments. Future research endeavors should aim to address these limitations by expanding the sample size, incorporating multicenter collaborations for validation, and conducting more in‐depth analyses to enrich the understanding of the subject matter.

In addition, although external validation was performed using an expanded cohort (*n* = 73), the sample size remains moderate and may limit the precision and generalizability of the model. Furthermore, the calibration intercept was not reported due to the unavailability of individual predicted probabilities, which may restrict a comprehensive assessment of model calibration. Larger, multicenter validation studies are warranted to further confirm the robustness of the findings.

While bootstrap resampling techniques were considered to assess performance variability, resampling within a limited external cohort does not introduce new population heterogeneity and may artificially reduce variance estimates. Therefore, we prioritized transparent reporting of CIs rather than relying solely on internal resampling techniques. Future studies should validate the present model in larger, multicenter cohorts across different geographic regions to further establish its robustness, reproducibility, and clinical applicability.

## 5. Conclusion

Our study presents a ML‐based binary prognostic model designed to distinguish between survival and mortality outcomes in patients with SFTS using routinely available laboratory indicators obtained at initial presentation. From a clinical integration perspective, this model has the potential to be incorporated into hospital laboratory information systems (LISs) or EHR platforms, enabling automated risk estimation within the first 24 h of admission.

Given its high sensitivity and NPV, the model may serve as an effective early triage tool to identify patients at high risk of mortality who require closer monitoring, intensive supportive care, or early transfer to higher‐level medical facilities.

By facilitating early identification of patients at high risk of mortality and supporting timely intervention, this integrated approach may improve clinical management, optimize resource allocation, and ultimately reduce mortality in SFTS.

## Author Contributions

Siqi Li wrote the scripts. Rujia Chen, Yun Wang, and Wei collected data from patient records and performed data entry. Renren Ouyang, Yun Wang, and Ting Wang performed statistical analyses. Siqi Li and Hongyan Hou designed and implemented the ML methodology and constructed and evaluated the ML model. They structured and evaluated ML models and drafted relevant methodological sections on feature selection, model building, and analysis. They also contributed to methodology sections on feature selection, model building, evaluation, and statistical analysis. Feng Wang, Hongyan Hou, and Shiji Wu analyzed the data and helped to revise the manuscript.

## Funding

This study was funded by the project of the National Natural Science Foundation of China (Grant No. 82372324).

## Ethics Statement

The questionnaire and methodology for this study was approved by the Human Research Ethics Committee of Tongji Hospital, Tongji Medical College, Huazhong University of Science and Technology (TJ‐IRB20230632), and the study was conducted in accordance with the Declaration of Helsinki.

## Consent

Informed consent was obtained from all individual participants included in the study. All authors contributed to the article and approved the submitted version.

## Conflicts of Interest

The authors declare that they have no conflicts of interest.

## Data Availability

All data generated or analyzed during this study are included in this published article.
